# Possible Phenotypic Consequences of Structural Differences in Idic(15) in a Small Cohort of Patients

**DOI:** 10.3390/ijms20194935

**Published:** 2019-10-05

**Authors:** Márta Czakó, Ágnes Till, András Szabó, Réka Ripszám, Béla Melegh, Kinga Hadzsiev

**Affiliations:** 1 Department of Medical Genetics, Medical School, University of Pécs, H-7624 Pécs, Hungary; till.agnes@pte.hu (Á.T.); szabo.andras@pte.hu (A.S.); ripszam.reka@pte.hu (R.R.); melegh.bela@pte.hu (B.M.); hadzsiev.kinga@pte.hu (K.H.); 2 Szentágothai Research Centre, H-7624 Pécs, Hungary

**Keywords:** idic(15) syndrome, array CGH, supernumerary marker chromosome, *GABA_A_* gene cluster, *CHRNA7*, epilepsy, autism spectrum disorder

## Abstract

Among human supernumerary marker chromosomes, the occurrence of isodicentric form of 15 origin is relatively well known due to its high frequency, both in terms of gene content and associated clinical symptoms. The associated epilepsy and autism are typically more severe than in cases with interstitial 15q duplication, despite copy number gain of approximately the same genomic region. Other mechanisms besides segmental aneuploidy and epigenetic changes may also cause this difference. Among the factors influencing the expression of members of the *GABA_A_* gene cluster, the imprinting effect and copy number differences has been debated. Limited numbers of studies investigate factors influencing the interaction of *GABA_A_* cluster homologues. Five isodicentric (15) patients are reported with heterogeneous symptoms, and structural differences of their isodicentric chromosomes based on array comparative genomic hybridization results. Relations between the structure and the heterogeneous clinical picture are discussed, raising the possibility that the structure of the isodicentric (15), which has an asymmetric breakpoint and consequently a lower copy number segment, would be the basis of the imbalance of the *GABA_A_* homologues. Studies of *trans* interaction and regulation of *GABA_A_* cluster homologues are needed to resolve this issue, considering copy number differences within the isodicentric chromosome 15.

## 1. Introduction

A wide variety of constitutional supernumerary marker chromosomes (SMCs) have been described in the human genome, with birth prevalence of 2–7 per 10,000 [1]. The identification and characterization of them became more and more accurate as the investigation methods evolved over time, which allowed for a more precise phenotype association and had many benefits in genetic counselling and prenatal diagnosis [2]. About half of the SMCs in humans are of 15 origins due to instability of 15q11.2–q13 genomic region. In the background of frequent rearrangements, there are five clusters of low copy repeats which are the basis of recurrent breakpoints known as BP1–BP5 being detected in the derivative chromosomes arising through different recombination events (non-allelic homologous recombination). U-type exchange is one of the crossovers which can result in a supernumerary isodicentric chromosome 15 (idic(15)) showing remarkable structural heterogeneity [3]. The most often described breakpoints involved in large idic(15) are BP4 and BP5 with two extra copies of genomic regions between BP2-BP3 (partial tetrasomy) [4]. The appearance of idic(15) can be also bisatellited; one of the centromeres can be inactive (pseudo-isodicentric form). It is of maternal origin in 70% of the patients and occurs with increasing frequency with advanced maternal age [5]. The clinical features characteristic for idic(15) are central hypotonia in early age, developmental delay (DD), moderate to severe intellectual disability (ID), seizures, autistic behaviour, and absent or very poor speech in the vast majority of the patients [6]. Major malformations are rarely reported. Incidence at birth is estimated to be 1 in 30,000 with a sex ratio of almost 1, however its incidence is probably higher due to under-ascertainment [7]. Karyotyping is not indicated in some patients due to subtle dysmorphic signs and the lack of major malformations, so they can remain undiagnosed. A high prevalence of idic(15) chromosome, out of the SMCs, is detected by fluorescence in situ hybridization (FISH) that targets the Prader–Willi/Angelman region of idic(15) as the next diagnostic procedure. After karyotyping, array comparative genomic hybridization (array CGH) is a powerful method for detailed characterization of the genomic content involved in idic(15), possibly providing additional data for genotype–phenotype correlation, especially in cases with atypical clinical signs. Here we report five idic(15) cases diagnosed at different ages, where the diagnosis was confirmed and completed by array CGH in each case.

## 2. Results

### 2.1. GTG Banding and FISH

**Figure 1:**
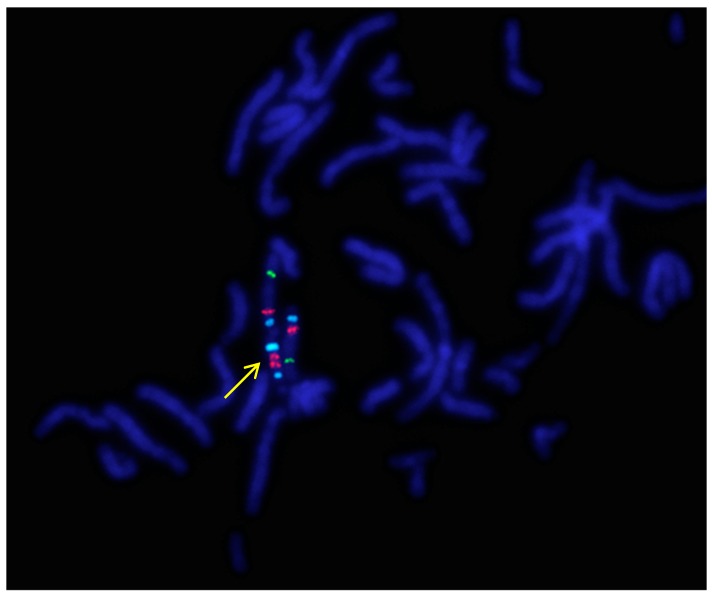
A representative metaphase fluorescence in situ hybridization (FISH) result of P.1. using the Prader–Willi/Angelman Critical Region (*UBE3A*) probe (Vysis, Abbott) (1000× magnification). The arrow indicates the idic(15). Signals: Aqua/blue—*D15Z1*, Orange/red—*UBE3A* locus, FITC/green—*PML* locus (15q24). Because of the signal positions, these probes show no difference between the markers of the five patients.

GTG-banding of the five patients (P.1.–P.5.) was performed at a 550-band level resolution. The karyotype of each patient contained a supernumerary acrocentric marker chromosome of G-group size. Based on the evaluation of 100 cells in each case, mosaicism could be excluded. Due to the clinical symptoms and the presence of the SMC, metaphase FISH analysis of the *UBE3A* locus (Prader–Willi/Angelman Critical Region) was performed in each case that showed the presence of both, the *D15Z1* and *UBE3A* regions on the SMCs (in addition to the normal chromosomes 15), thereby confirming that the extra chromosome is of 15 origin (Figure 1).

In details, the karyotypes of Patient 1.–Patient 5. can be described and summarized as 47, XN, +idic(15) (pter→q13::q13→pter) (100). Each abnormality resulted in tetrasomy of the 15q11q13 region. None of the patients was mosaic. Parental cytogenetic studies were normal.

### 2.2. Uniparental Disomy

Uniparental disomy could be excluded only in Patient 2. and 3. The parents of Patients 1., 4. and 5. did not consent to the study.

### 2.3. Array CGH

The copy numbers and breakpoints of the different genomic regions in the SMCs 15 are presented in Table 1. (according to ISCN 2016) [8]. In addition, common benign variants were detected. The base pair positions of the genomic imbalances were designated according to the February 2009 Assembly (GRCh37/hg19).

## 3. Discussion

There is a wide range of severity in the clinical signs experienced by individuals with idic(15) syndrome. Various genetic mechanisms have been hypothesized to explain this clinical heterogeneity. Only one of the five examined patients (P.1.) has a SMC which shows a characteristic symmetrical structure with a single breakpoint. This formation mechanism resulted in the presence of four copies uniformly throughout the segmental aneuploidy. Unlike the others, this patient showed no symptoms of epilepsy or autism up to 20 months of age. However, it must be pointed out that there is a possibility he may develop epilepsy and/or autism at a later point. In the course of patient management, careful monitoring of seizure and behaviour is warranted in this case.

Several theories have been published analysing the relationship between epilepsy observed in idic(15) patients and the genes involved in the supernumerary chromosome. Some of them highlighted the extra copy numbers of the affected genes, others the various mechanisms leading to altered gene expression, as well as imprinting as possible causes of seizures. The electro-clinical phenotype, course, and response to therapy of epilepsy are very heterogeneous in idic(15), despite the presence of the extra marker chromosome as common causative factor [9,10]. As it has become apparent, differences in the breakpoints and affected genomic regions do not provide sufficient explanation for the observed clinical heterogeneity. Earlier studies of similar patients raised the possibility that extra copies of the 15q11.2–13 region may cause an imbalance of homologous pairing of the alleles involved in segmental aneuploidy [3,11,12]. This may explore another mechanism in this complex region containing several imprinted and biallelically expressed genes leading to the formation of an abnormal phenotype. The cluster of three GABA_A_ receptor subunit genes (*GABRB3, GABRA5*, and *GABRG3* which encode the receptor subunits β3, α5, and γ3, respectively) is of particular importance for neurodevelopmental disorders with epilepsy and autism because of GABA (Gamma amino-butiric acid) being the main inhibitory neurotransmitter in the brain [13]. Hogart et al. suggested that *trans* interactions between 15q11–13 paternal and maternal homologues may have significance in optimal biallelic expression of the genes within the GABA_A_ receptor domain [11]. Deficiency or reduced level of GABRB3 protein is associated with seizures, sleep disturbances, and stereotyped behaviours in mice [14]. Furthermore, significant GABRB3 reduction was observed in Rett syndrome and Angelman syndrome as well. The EEG patterns characteristic for Angelman and Rett syndrome patients, respectively, also support the role of *GABRB3* in the formation of epileptic phenotype [15,16]. Hogart et al. demonstrated that impairment of the biparental chromosomal contribution has a negative effect on *GABRB3* expression [11]. Based on these data, it is thoughtful that the phenotype of the four patients reported here with both epilepsy and autistic features may be related to the asymmetric marker structure. Among the possible pathomechanisms leading to the symptoms, one should also consider that the lower copy number of a section of the marker interferes with the *trans* interaction of homologues originating from the two parents. 

Furthermore, the possible role of the *CHRNA7* gene on autism and epilepsy has repeatedly emerged in the literature; however, its clinical significance is debated to this day [17]. Analysis of cases with different size of microduplications involving *CHRNA7*—which encode the alpha 7 subunit of nicotinic acetylcholine receptors—rather suggests that it might represent a risk factor for neurobehavioral disorders. With regard to our patients, *CHRNA7* was present in the idic(15) chromosome of all patients with exception of P.1., the only one who has neither seizures nor autistic features. This patient has an idic(15) in which *CHRNA7* is not involved.

Types of seizures observed in our patients, their EEG abnormalities, the age at the first seizure, the defined electro-clinical epilepsy syndrome, and the response given to anti-epileptic drugs (Table 2) did not differ from the previously published data [9,10]. The management of epilepsy is the most difficult medical challenge in 75% of patients with idic(15) syndrome [6]. To date, four studies have provided data on a greater number of patients regarding the course and treatment of their seizures. Besides the above mentioned [9,10], Conant et al. reported 83 idic(15) cases; 53 of them had epilepsy, in 81% multiple seizure types occurred, and 42% had infantile spasms [18]. Similar to our patients, further described seizures were of tonic-clonic, atonic, myoclonic, and focal types. In another study of 35 idic(15) patients, Battaglia et al. reported characteristics of epilepsy (28/35 cases). Epilepsy was well controlled in 32% of the analysed cases, satisfactory controlled in 10.7%, and uncontrolled in 32% despite polytherapy [19]. According to the literature, drug-resistant epilepsy occurs in many cases evolving into LGS [20,21]. Among our patients there are two subjects with identical, asymmetrical breakpoints (P.2. and P.3.) whose epilepsy started in infancy with epileptic spasms leading to the electro-clinical diagnosis of WS. The severity of their DD/ID is much more pronounced than in the other three cases with different molecular mechanisms. Matricardi et al. demonstrated a link between the age of seizure onset and the long-term outcome of epilepsy, with a more severe prognosis when seizures started early [10]. In order to determine whether there is a causative relationship between the mechanism of idic(15) formation, the resulting asymmetric structure, and the more severe phenotype, detailed investigation of several patients with a molecular subtype similar to P.2. and P.3. would be necessary.

ID/DD affects around 2–4% of individuals in different populations worldwide. It is a severe condition that the patient needs lifelong care in different levels, due to lack of definitive therapy. Establishing an accurate diagnosis is essential in developing an accurate therapy in the future. Thanks to the introduction of new generation examination techniques in the field of clinical genetics, the number of unidentified cases reduced significantly in the last decades [22,23,24]. Early identification of idic(15) syndrome is beneficial for providing early and appropriate intervention through careful attention to the development of seizure and autism.

## 4. Materials and Methods 

### 4.1. GTG Banding

Karyotyping was carried out by Giemsa–Trypsin (GTG) banding from peripheral blood lymphocytes using standard procedures [25]. In order to exclude mosaicism, 100 cells were analysed in each case. 

### 4.2. FISH

The *UBE3A* locus (Prader–Willi/Angelman Critical Region) specific probe was applied for FISH examination containing controls in 15p11.2 and 15q22 regions, respectively (Vysis, Abbott Laboratories, Abbott Park, IL, USA) [26]. The protocol used was in accordance with the manufacturer’s instructions.

### 4.3. Uniparental Disomy

Uniparental disomy of chromosomes 15 was investigated in Patient 2. and 3. using polymorphic STR markers: D15S11, D15S122, D15S128, D15S210, D15S97, D15S113, GABRB3, D15S165, and D15S659, respectively [27,28]. After PCR amplification of the markers, the resulting products were separated on a polyacrylamide gel and detected by silver staining [29]. The parents of the other three patients did not consent to the study. 

### 4.4. Array CGH

Array CGH was performed using Agilent Human Genome Unrestricted G3 ISCA v2 Sureprint 8x60K oligo-array (Amadid 021924; it is a microarray with high resolution containing 18,851 60-mer oligo probes in ISCA regions (International Standards for Cytogenomic Arrays Consortium) and 40,208 backbone probes with an average 60 KB overall median probe spacing in coding and non-coding genomic regions, respectively) (Agilent, Santa Clara, CA, USA) [30]. DNA was purified from peripheral blood using the NucleoSpin^®^Dx Blood DNA Purification Kit (Thermo-Fisher Scientific, Waltham, MA, USA) according to the protocol of the manufacturer. NanoDrop spectrophotometer was applied for calculation of the concentration and purity of the isolated DNA. Labelling and hybridization of the samples was prepared according to the Agilent Oligonucleotide Array-Based CGH for Genomic DNA Analysis—Enzymatic Labelling Protocol. The patient’s DNA and a sex-matched reference DNA (1 μg from each) were digested with *Alu*I and *Rsa*I enzymes for 2.5 h at 37 °C. The digested DNA was labelled via random priming (Agilent Genomic DNA Labelling Kit; Agilent, Santa Clara, CA, USA) using Cy5-dUTP for patient samples and Cy3-dUTP for control DNA, respectively. Purification after labelling was performed by Amicon Ultra AU-30 filters. The patient and reference samples with 50 μg Human Cot-1 DNA together were cohybribized at 65 °C for 24 h. Washing was performed following the instructions of Agilent Protocol v7.2. Array image was obtained by Agilent dual laser scanner G2565CA and analysed with Agilent Feature Extraction software (v10.10.1.1.). Agilent Cytogenomics software (v2.5.8.11) was used for visualization of the results. DNA sequence information refers to the public UCSC (University of California, Santa Cruz, Genome Browser) database. The copy number variations detected were compared to known aberrations available in public databases like DECIPHER (Database of Chromosomal Imbalance and Phenotype in Humans using Ensembl Resources), the Database of Genomic Variants, Clingen Dosage Sensitivity Map, ClinVar, and Ensembl (Ensembl GRCh37 Release 97 (July 2019)).

### 4.5. Case Presentation

#### 4.5.1. Patient 1.

Patient 1. was born from his mother’s twin pregnancy by caesarean section at 37 weeks of gestation with a birth weight of 2810 g (10–25 percentile). His twin-sister is healthy. Family history was unremarkable. Severe generalized hypotonia was observed from birth; later DD became evident. Metabolic evaluation (that included urine organic acids, serum amino acids, and thyroid function tests) was normal. Magnetic resonance imaging (MRI) of the brain showed normal intracranial morphology. Cardiological, ophthalmological, and audiological examinations showed normal results. At the age of 1 year he was referred to genetic evaluation. His weight was 8770 g (10–25 percentile), his height was 77 cm (50–75 percentile), and head circumference was 46.5 cm (50–75 percentile). He had asymmetric skull, flat occipital region, hypertelorism, epicanthal folds, broad nose, prominent philtrum, and wide lips. Neurological examination revealed severe generalized hypotonia with normal deep tendon reflexes and absent speech development, his developmental quotient evaluated at 1 year of age was 46.8 (Brunet–Lezine test). Epilepsy has not been noted so far and electroencephalography (EEG) gave negative result. At the age of 18 months he could sit alone, roll over; he could reach to get a toy with palmar grasp. He started to babble; he was able to share enjoyment and to make eye contact. Stereotyped movements were not observed (Table 3).

#### 4.5.2. Patient 2.

Patient 2. was born by normal spontaneous vaginal delivery at 41 weeks of gestation with a birth weight of 3520 g (75–90 percentile). She is the second child of non-consanguineous young couple. Family history was unremarkable. Developmental milestones were normal at early age: head control and rolling over at 5 months, but thereafter, DD became evident. She had infantile spasms at 8 months; EEG showed hypsarrhytmia and burst suppression during sleep. The diagnosis of West syndrome (WS) was made and vigabatrin therapy was administered, which caused significant seizure frequency reduction (Table 3). Brain MRI showed symmetrically widened extracerebral liquor spaces in the frontal and temporal regions. At the age of 17 months she was referred to genetic evaluation, her weight was 13 kg (90–97 percentile), her height was 85 cm (90–97 percentile), and head circumference was 46 cm (50 percentile). Her facial dysmorphic features were subtle: flat occipital region; wide face; prominent forehead and small, turned-up nose; broad thorax; and small feet could be observed. Neurological examination revealed moderate hypotonia with normal reflexes and global developmental delay. She could not walk alone; her expressive language development was delayed as well, and only babbling was present. She had problems with chewing and swallowing. At that time, she showed autistic behaviours including stereotypic movements like hand-clapping, hand-shaking, as well as self-swinging.

#### 4.5.3. Patient 3.

Patient 3. was the second child of healthy, non-consanguineous parents. Family history was unremarkable. He was born by spontaneous vaginal delivery at 40 weeks of gestation with a birth weight of 3200 g (25–50 percentile). Severe generalized hypotonia was observed from birth and his developmental milestones were delayed. He had feeding difficulties in infancy. The first epileptic seizure (infantile spasms) developed at 6 months of age. WS has evolved later into therapy-resistant Lennox–Gastaut syndrome (LGS), various seizure types appeared (Table 3). Brain MRI showed normal intracranial morphology. At the age of 27 months, he was referred to genetic evaluation, his weight was 10,400 g (<3 percentile), his height was 90.5 cm (50–75 percentile), and his head circumference was 46 cm (3 percentile). He had flat occipital region, hypertelorism, epicanthal folds, oesotropia, broad and flat nasal bridge, and crease of earlobes. Neurological examination indicated severe hypotonia, global DD and autistic behaviour. He could not walk alone; he could not chew and could not say meaningful words at that time.

#### 4.5.4. Patient 4.

Patient 4. was the first child of a healthy young couple. He was born by spontaneous vaginal delivery at 41 weeks of gestation with a birth weight of 4100 g (90–97 percentile). His postnatal adaptation was uneventful. His developmental milestones were delayed. He suffered from permanent diarrhoea from birth, but gastroenterological examination did not detect any obvious reason of it. His first epileptic seizure developed at the age of 5 years; he had generalized tonic-clonic, atonic, and myoclonic seizures as well. His epilepsy was proved to be therapy-resistant; the best seizure control was achieved by lamotrigine monotherapy (Table 3). Brain MRI showed periventricular leukomalacia with mild atrophy of the corpus callosum. He was referred to genetic evaluation at the age of 7 years; his weight was 22 kg (25–50 percentile), his height was 119 cm (10–25 percentile), and he had microcephaly; his head circumference was 49 cm (<3percentile). Physical examination revealed mild axial hypotonia with spastic paraparesis and mild facial dysmorphisms, such as prominent ears and thin and soft hair with low-set hairline. He could walk with orthesis but his walking was unstable. He could speak short sentences but started to stutter. Autistic behaviour was characteristic of him, bruxism, hand-shaking, and whirling was permanently observed.

#### 4.5.5. Patient 5.

Patient 5. was born at term by caesarean section with a birth weight of 3050 g (25–50 percentile) after an uneventful pregnancy. There were no problems with her adaptation in the perinatal period. Her older sister is healthy, but her father died of sudden cardiac death at the age of 36. She could walk alone at 18 months of age, but her walking was always very unstable. She started to talk words in time, but later her speech became very simple and dyslalic. Autistic behaviour and ID became evident in early childhood, and she could start school in a special class only. The first epileptic seizure developed at 8 years of age as an atypical absence seizure. Later various seizure types occurred, and her epilepsy evolved into a therapy-resistant LGS (Table 3). Brain MRI gave normal result. She was referred to genetic evaluation at the age of 8; her weight was 44 kg (above 97 percentile); her height was 138 cm (90–97 percentile), and her head circumference was 53 cm (90–97 percentile). Besides the obesity due to overeating, the physical examination revealed mild generalized hypotonia and subtle dysmorphic facial features like long face, epicanthal folds, long philtrum, small mouth, thick lower lip, and high-arched palate.

### 4.6. Ethics Approval

Written informed consent was obtained from all subjects. The collection and usage of DNA samples and management of data followed the Helsinki Declaration of 1975 and was in accordance with the Hungarian law (XXI/2008) for genetic examination, research and biobanking. The study design was approved by the HRB National Ethics Committee (17 February 2017).

## 5. Conclusions

Idic(15) should be investigated in patients presenting difficult to treat epilepsy such as WS and LGS without a structural brain lesion and in patients with early central hypotonia even in the absence of dysmorphic features.

Array CGH is a powerful method for detailed characterization of the genomic content involved in idic(15), even if the chromosomal aberrations were previously detected. Since this technique provides valuable information on the copy number of each section involved in idic(15), its use can contribute to the better understanding of the genotype–phenotype relationship. The exact knowledge of the marker’s composition is important for the clinical management of the patient as well.

## Figures and Tables

**Figure 2:**
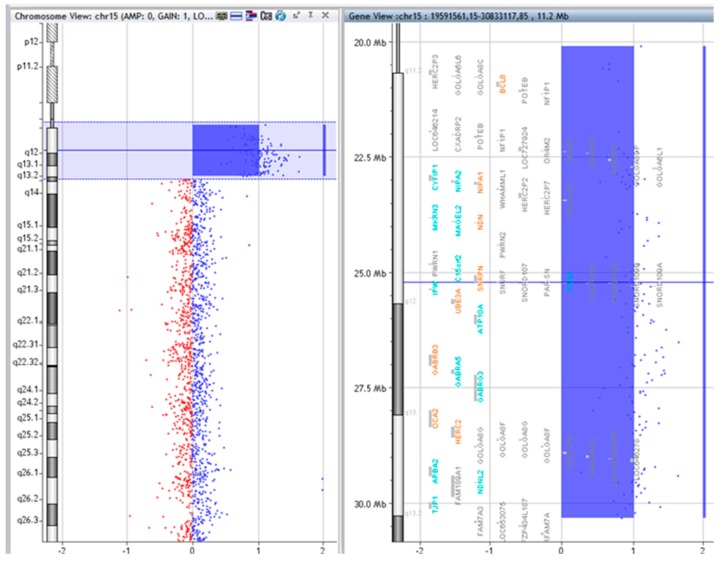
Array comparative genomic hybridization (CGH) results of P.1.

**Figure 3:**
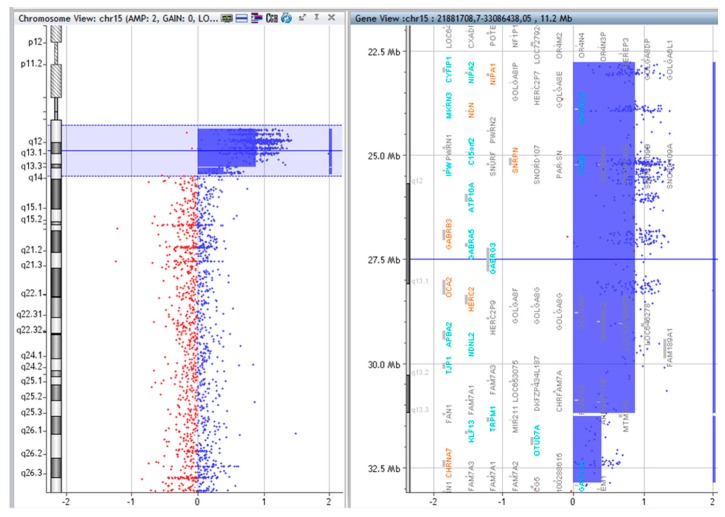
Array CGH results of P.2.

**Figure 4:**
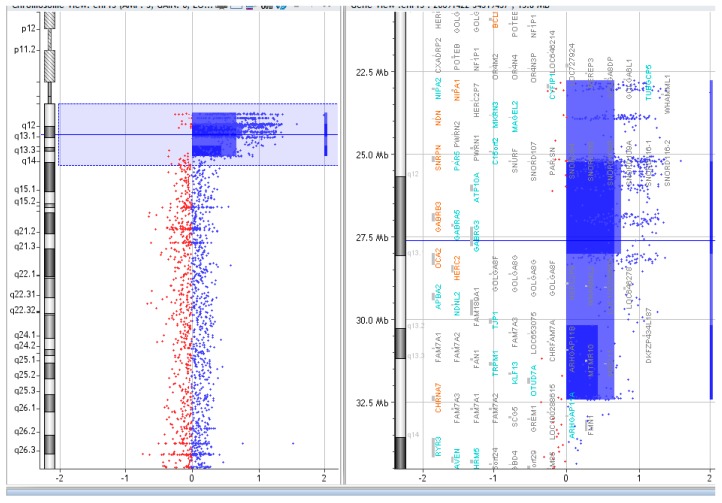
Array CGH results of P.3.

**Figure 5:**
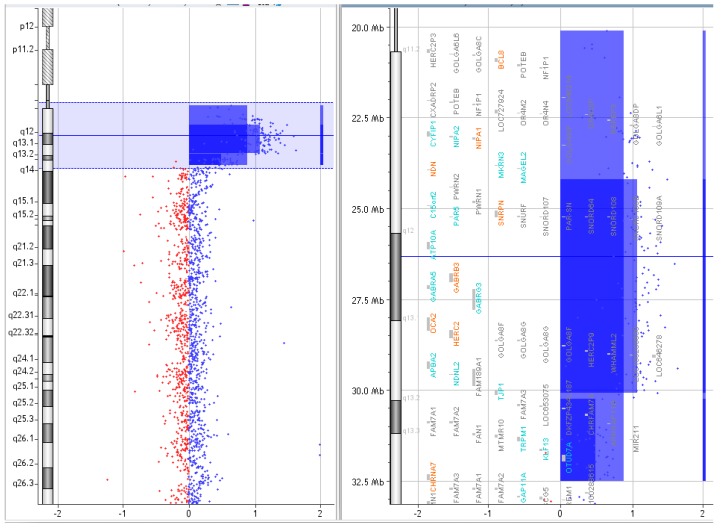
Array CGH results of P.4.

**Figure 6:**
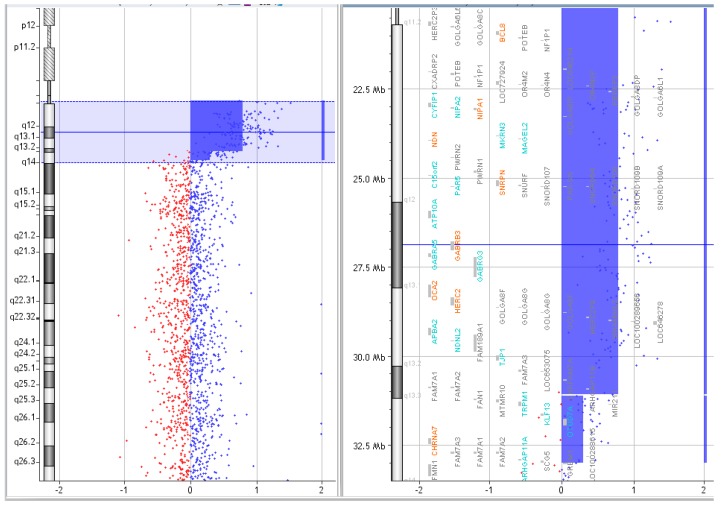
Array CGH results of P.5.

**Table 1:** Molecular subtype and array CGH results of the idic(15) chromosomes.

Patient	Molecular Subtype	Array CGH Results	Figures
P.1.	A	arr [GRCh37] 15q11.1q13.2(20102541_30322138)x4	Figure 2.
P.2.	C	arr [GRCh37] 15q11.2q13.2(22765628_31183907)x4, arr [GRCh37] 15q13.3(31261835_32861626)x3	Figure 3.
P.3.	C	arr [GRCh37] 15q11.2q13.3(22765628_30178222)x4, arr [GRCh37] 15q13.1q13.3(30226187_32445252)x3	Figure 4.
P.4.	D	arr [GRCh37] 15q11.1q13.3(20102541_30078386)x4, arr [GRCh37] 15q13.1q13.3(30251859_32510863)x3	Figure 5.
P.5.	B	arr [GRCh37] 15q11.1q13.2(20102541_31077833)x4, arr [GRCh37] 15q13.2q13.3(31123186_33009483)x3	Figure 6.

A. Large idic(15)—symmetrical breakpoints BP1-BP4:BP4-BP1. B. Large idic(15)—asymmetrical breakpoints BP1-BP5:BP4-BP1. C. Large idic(15)—asymmetrical breakpoints BP2-BP5:BP4-BP2. D. Large idic(15)—asymmetrical breakpoints BP1-BP5:BP3-BP1.

**Table 2:** Summary of epilepsy related data.

Patient	Epilepsy				
	Age at Onset	Type of Seizures	AEDs	Type of Epilepsy	Current Status
1.	-	-	-	-	seizure free
2.	8 m	infantile spasms	VGB	WS	seizure free from age 2 y
3.	6 m	infantile spasms, tonic, atonic, focal	FLB RFN	WS LGS	daily
4.	5 y	tonic-clonic	LVT LTG	LGS	monthly
5.	8 y	atonic, myoclonic	TPM VPA	LGS	daily

Abbreviations: y: year; m: month; WS: West syndrome, LGS: Lennox–Gastaut syndrome; VGB: vigabatrin; FLB: felbamate; RFN: rufinamide; LVT: levetiracetam; LTG: lamotrigine; TPM: topiramate; VPA: valproic acid.

**Table 3:** Summary of the patients’ main clinical symptoms.

Patient	Gender	Age at Diagnosis	Hypotonia	ID	Speech	Behaviour
1.	m	1 y	severe	moderate	no	-
2.	f	17 m	moderate	severe	no	stereotypies
3.	m	27 m	moderate	severe	no	ASD
4.	m	7 y	mild	moderate	short sentences	ASD
5.	f	8 y	mild	mild	sentences	ASD

Abbreviations: m: male; f: female; y: year; m: month; ID: intellectual disability, ASD: autism spectrum disorder.

## Abbreviations

array CGH	Array comparative genomic hybridization
ClinVar	Database of Clinical Variants
DD	Developmental delay
DECIPHER	Database of Chromosomal Imbalance and Phenotype in Humans using Ensembl Resources
EEG	Electroencephalography
FISH	Fluorescence in situ hybridization
GABA	Gamma amino-butiric acid
GRCh	Genome Reference Consortium Human Build 37
GTG banding	Giemsa-Trypsin banding
ID	Intellectual disability
idic(15)	Isodicentric chromosome (15)
ISCA	International Standards for Cytogenomic Arrays Consortium
LGS	Lennox–Gastaut syndrome
MRI	Magnetic Resonance Imaging
SMC	Supernumerary marker chromosome
UCSC	University of California, Santa Cruz, Genome Browser database
WS	West syndrome
